# Supplementary material for: Utargetome: A targetome prediction tool for modified U1-snRNAs to identify distal-target positions with improved selectivity
Source: PLoS Comput Biol. 2025 Sep 23;21(9):e1013534. doi: 10.1371/journal.pcbi.1013534 (PMC12527174; doi:10.1371/journal.pcbi.1013534)
Supplement: S1 Fig — (DOCX) [file pcbi.1013534.s001.docx]

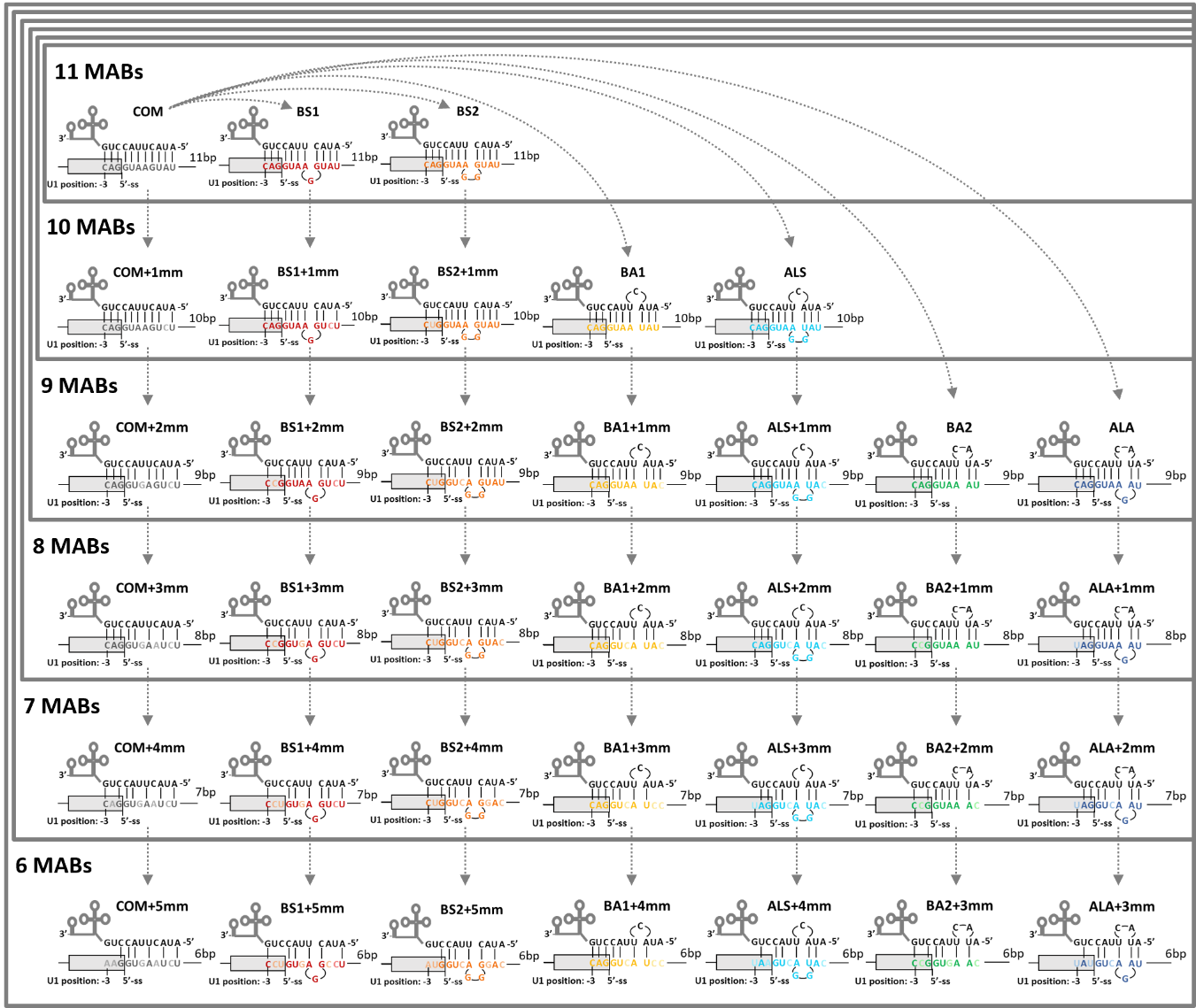


**S1 Fig.** Representation of targetome creation for a given U1 in the Utargetome pipeline. Starting with the fully complementary target of a given U1 (“COM”) and provided a specific number of MABs (minimum 4), the full targetome is built by 1) removing or inserting nucleotides in the target sequence to reproduce loops and bulges (curved arrows) and 2) gradually inserting mismatched positions (straight arrows) until the minimum number of base-pairs is reached (corresponding to the given MABs). Specifically: BS1 and BS2 (single- and double-nucleotide bulges on the RNA target strand) are implemented by inserting 1 and 2 nt respectively; BA1 and BA2 (single- and double-nucleotide bulges on the U1 strand) are implemented by deleting 1 and 2 nt respectively; ALS (asymmetric loops with the larger loop on the target RNA strand) are implemented by deleting 1 nt and inserting a new combination of 2 nt; ALA (asymmetric loops with the larger loop on the U1 strand) are implemented by deleting 2 nt and inserting 1 new nt. Base-pairing mismatches are subsequently implemented as new combinations of nucleotides, with at least one nucleotide apart from the budge(s)/loop (if present) to preserve their hypothetical structure. The position of a predicted target in reference to a nearby 5’- or 3’-SS always refers to the position of the 5’-most nucleotide on the target sequence, i.e. the 3’-most nucleotide on the U1 antisense sequence, regardless of whether the base-pairing consists of a canonical Watson-Crick or a mismatched pairing. All targets in the figure are located at position “-3” from the 5’-SS.
