# Supplementary material for: Utargetome: A targetome prediction tool for modified U1-snRNAs to identify distal-target positions with improved selectivity
Source: PLoS Comput Biol. 2025 Sep 23;21(9):e1013534. doi: 10.1371/journal.pcbi.1013534 (PMC12527174; doi:10.1371/journal.pcbi.1013534)
Supplement: S3 Fig — (DOCX) [file pcbi.1013534.s003.docx]

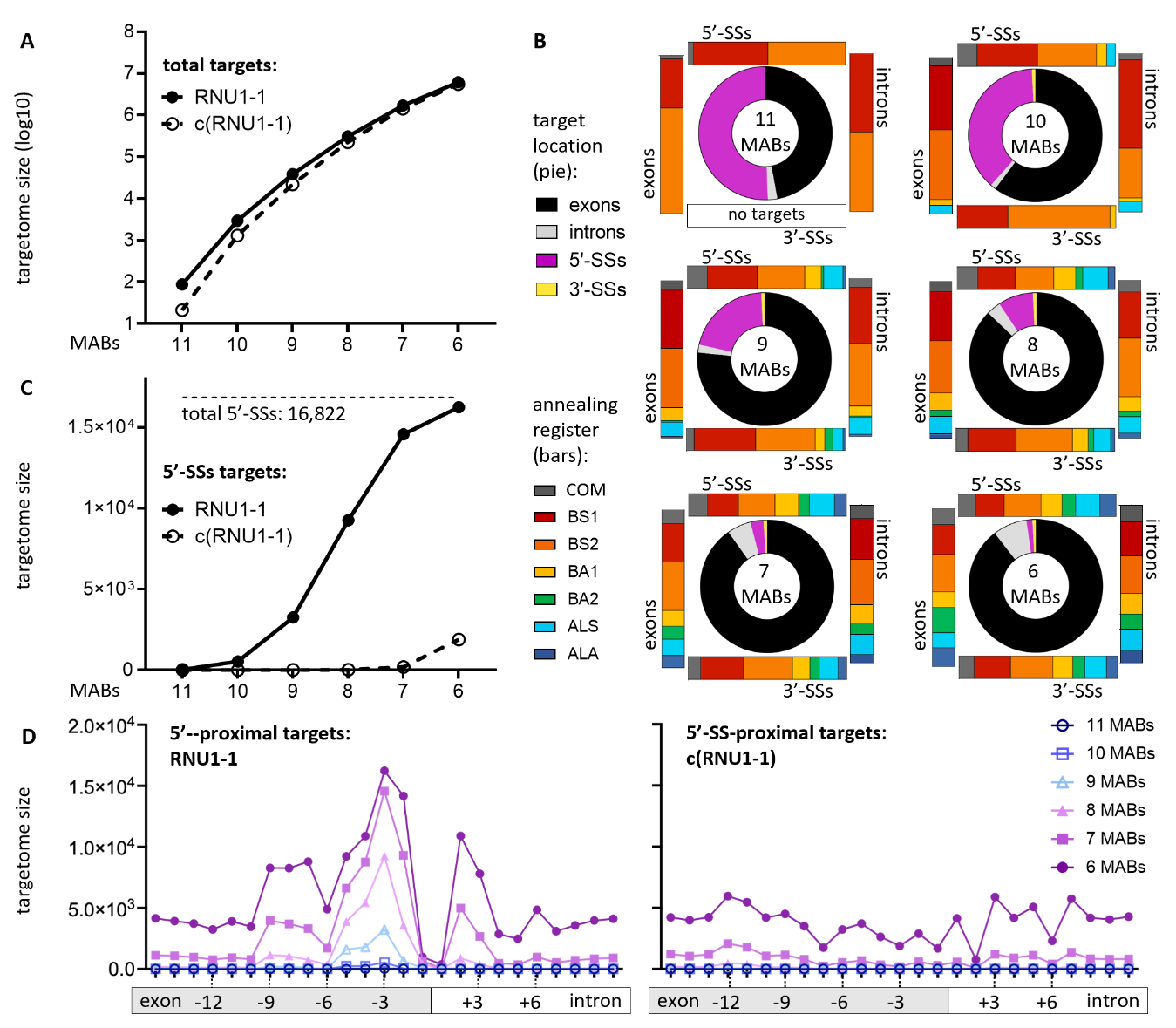


**S3 Fig.** Targetome of the endogenous U1 of *D. discoideum*. The 5’-terminal nucleotides of RNU1-1 transcript (5’-AUACUUACCUG-3’) were used as the input binding sequence. Targetome of its complementary sequence was analyzed as control. Refer to Fig 3 for the legend.
