# Supplementary material for: Utargetome: A targetome prediction tool for modified U1-snRNAs to identify distal-target positions with improved selectivity
Source: PLoS Comput Biol. 2025 Sep 23;21(9):e1013534. doi: 10.1371/journal.pcbi.1013534 (PMC12527174; doi:10.1371/journal.pcbi.1013534)
Supplement: S4 Fig — (DOCX) [file pcbi.1013534.s004.docx]

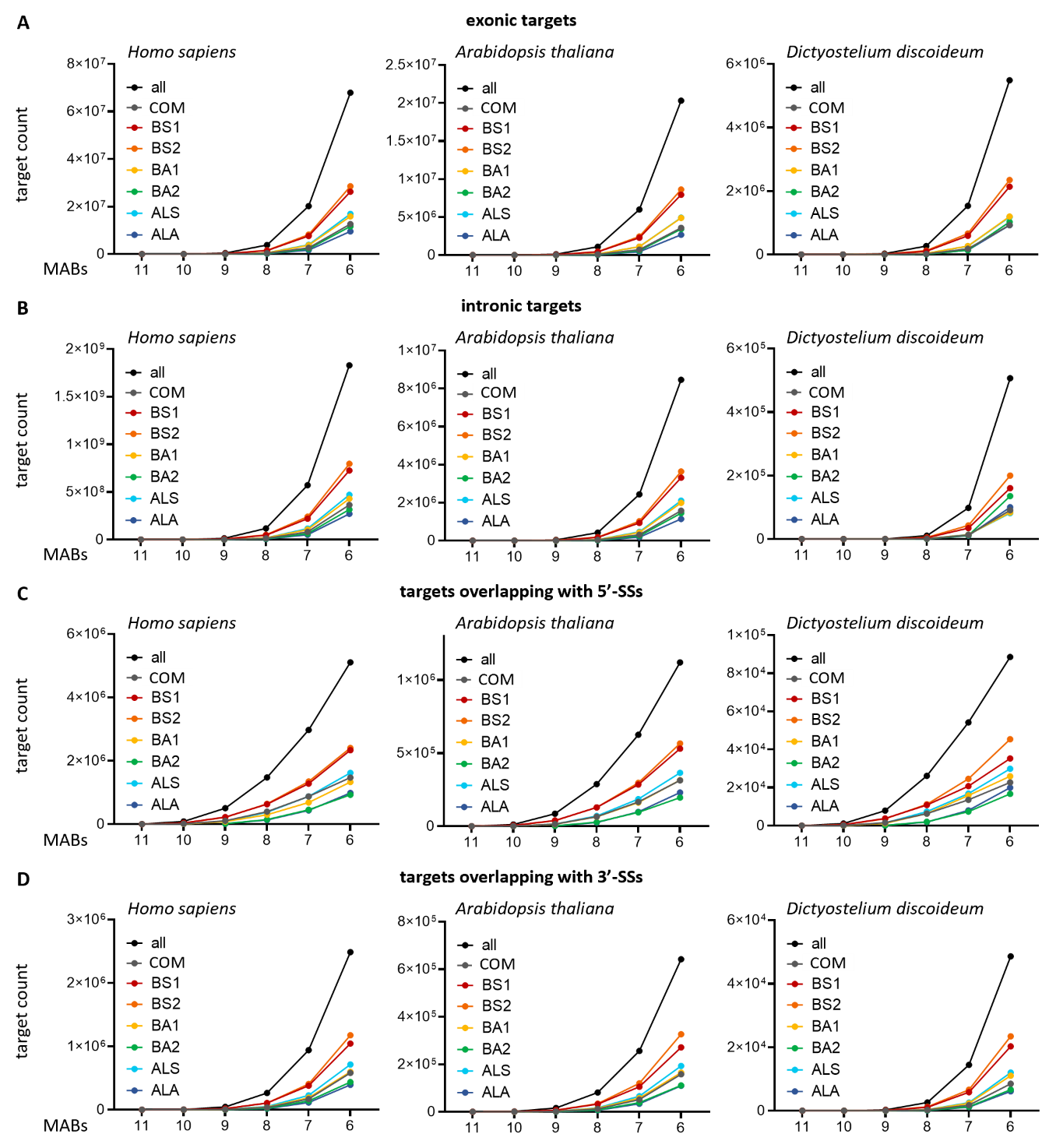


**S4 Fig.** Proportion of alternative annealing registers in the targetomes of the endogenous U1 in *H. sapiens*, *A. thaliana* and *D. discoideum*. Targets were considered from 11 to 6 MABs, at (**A**) exons, (**B**) introns, (**C**) overlapping with 5’-SSs or (**D**) 3’-SSs.
