# Supplementary material for: Utargetome: A targetome prediction tool for modified U1-snRNAs to identify distal-target positions with improved selectivity
Source: PLoS Comput Biol. 2025 Sep 23;21(9):e1013534. doi: 10.1371/journal.pcbi.1013534 (PMC12527174; doi:10.1371/journal.pcbi.1013534)
Supplement: S5 Fig — (DOCX) [file pcbi.1013534.s005.docx]

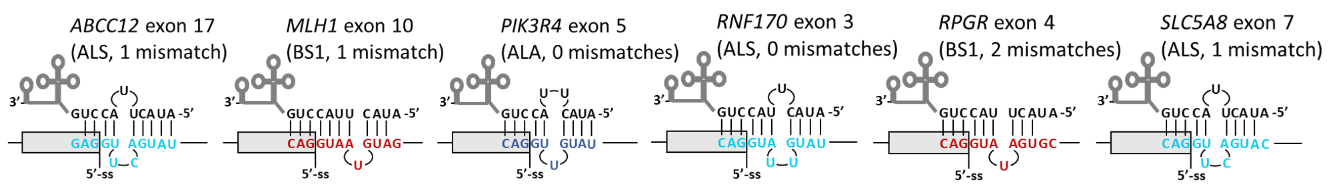


**S5 Fig.** Reported donor splice sites bound by the endogenous U1 through alternative annealing registers. From left to right: ALS with 1 mismatch at *ABCC12* exon 17 (ENSG00000140798, chr16:48108440-48108529); BS1 with 1 mismatch at *MLH1* exon 10 (ENSG00000076242, chr3:37017506-37017599); ALA with no mismatch at *PIK3R4* exon 5 (ENSG00000196455, chr3:130716396-130716599); ALS with no mismatch at *RNF170* exon 3 (ENSG00000120925, chr8:42873931-42874006); BS1 with 2 mismatches at *RPGR* exon 4 (ENSG00000156313, chrX:38321027-38321089); ALS with 1 mismatch at *SLC5A8* exon 7 (ENSG00000256870, chr12:101187386-101187515).
