# Supplementary material for: Utargetome: A targetome prediction tool for modified U1-snRNAs to identify distal-target positions with improved selectivity
Source: PLoS Comput Biol. 2025 Sep 23;21(9):e1013534. doi: 10.1371/journal.pcbi.1013534 (PMC12527174; doi:10.1371/journal.pcbi.1013534)
Supplement: S6 Fig — (DOCX) [file pcbi.1013534.s006.docx]

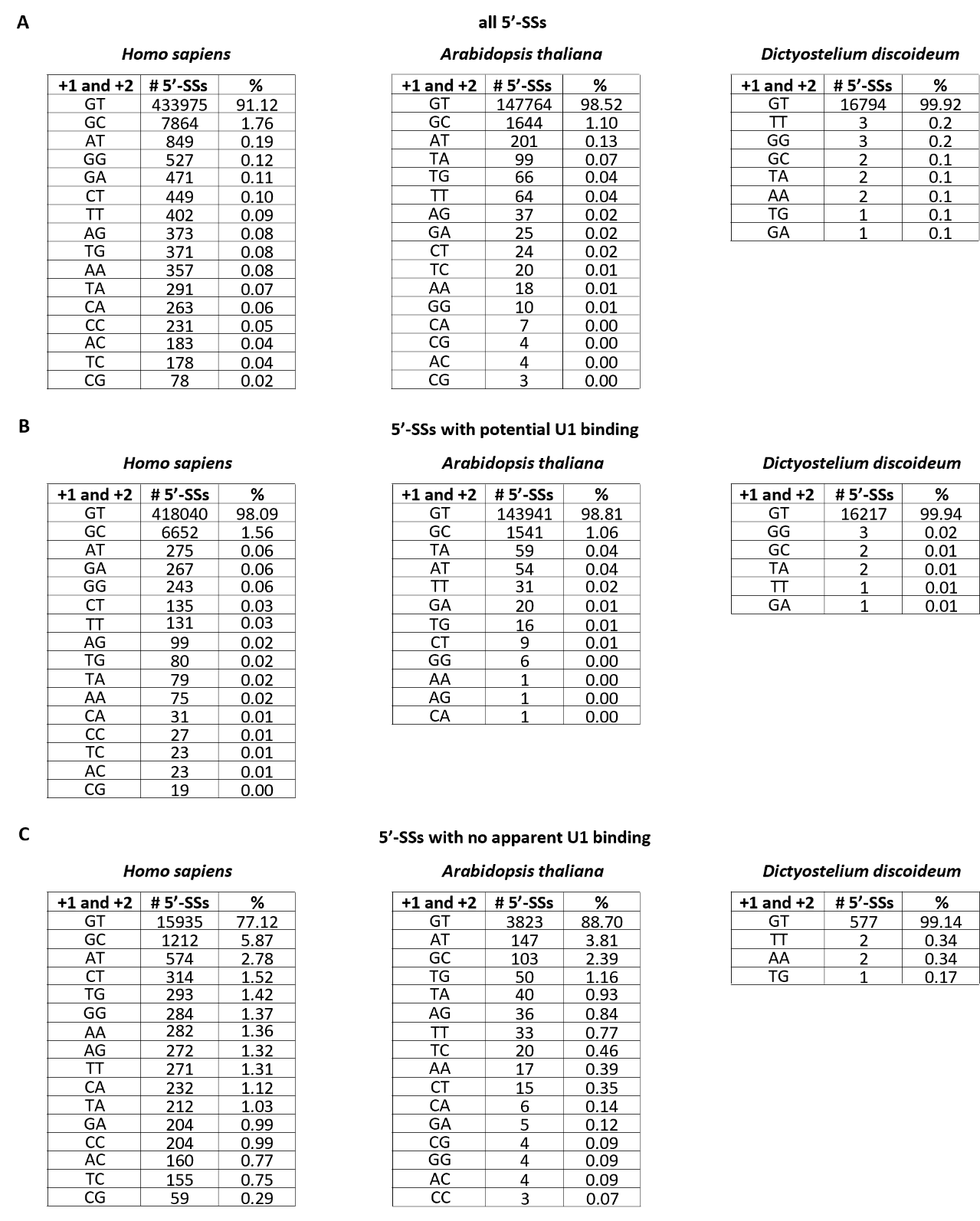


**S6 Fig.** Dinucleotide combinations at positions +1 and +2 of 5’-SS targets of the endogenous U1 in *H. sapiens*, *A. thaliana* and *D. discoideum*. The number of 5’-SSs carrying each dinucleotide combination (“# 5’-SSs”), and their percentage in the total count (“%”) are shown for: (**A**) all annotated 5’-SSs; (**B**) potential U1 binding sites with 6 MABs at the canonical position; (**C**) sites with no predicted U1 binding site (i.e. all 5’-SSs that have not been matched by the endogenous U1 with 6 MABs).
