# Supplementary material for: Utargetome: A targetome prediction tool for modified U1-snRNAs to identify distal-target positions with improved selectivity
Source: PLoS Comput Biol. 2025 Sep 23;21(9):e1013534. doi: 10.1371/journal.pcbi.1013534 (PMC12527174; doi:10.1371/journal.pcbi.1013534)
Supplement: S7 Fig — (DOCX) [file pcbi.1013534.s007.docx]

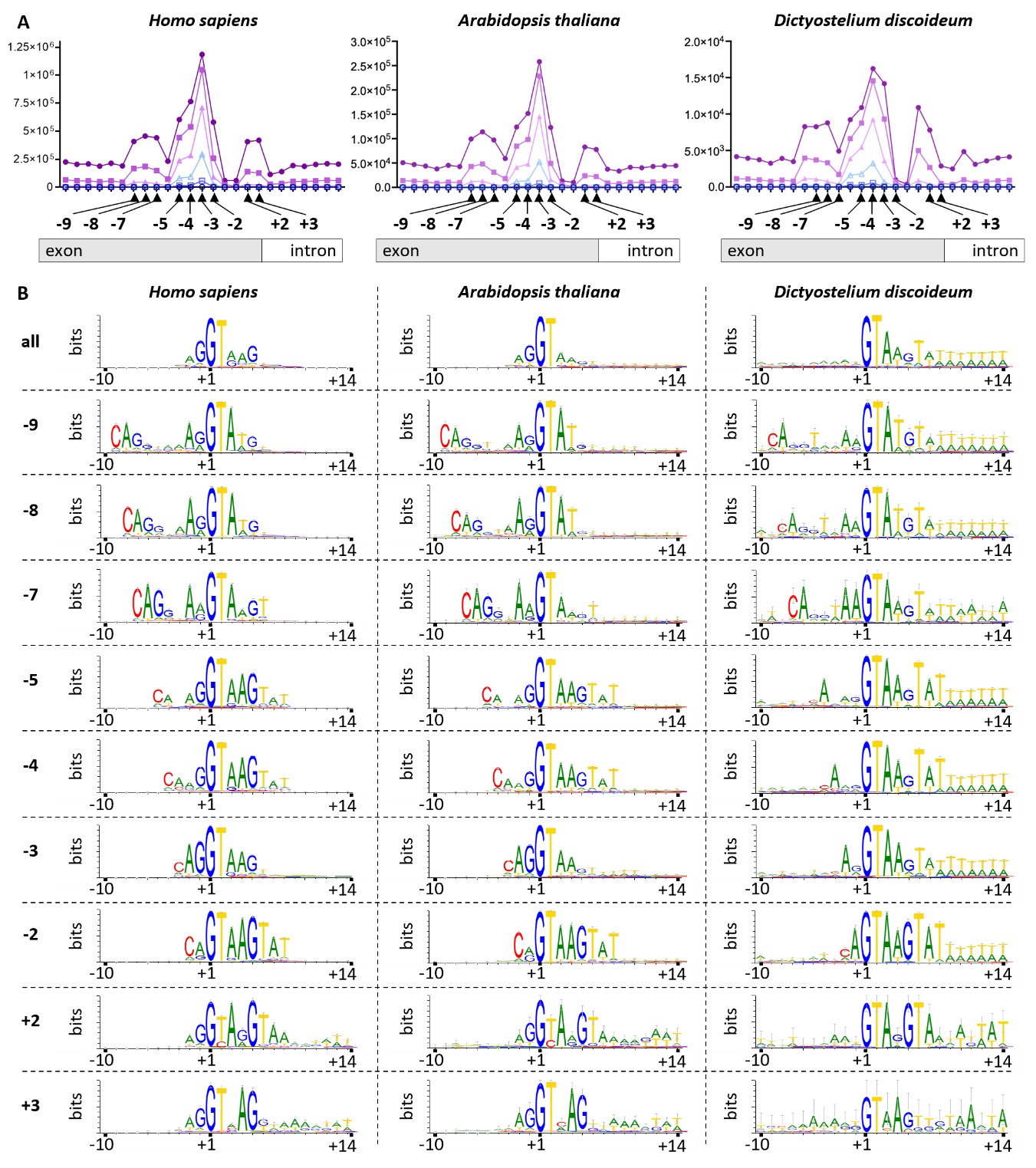


**S7 Fig.** Potential binding motifs of the endogenous U1 at distal positions in *H. sapiens*, *A. thaliana* and *D. discoideum*. (**A**) Distribution of endogenous U1 targets in proximity of 5’-SSs is shown for all 3 species as in Fig 3D, S3D and S4D. Positions of interest are indicated by black arrows. (**B**) WebLogo PWMs, generated from target sequences with 9 MABs, are shown for the positions of interest. Positions on the horizontal axis of the matrices indicate the distance from the splice site.
