# Supplementary material for: Utargetome: A targetome prediction tool for modified U1-snRNAs to identify distal-target positions with improved selectivity
Source: PLoS Comput Biol. 2025 Sep 23;21(9):e1013534. doi: 10.1371/journal.pcbi.1013534 (PMC12527174; doi:10.1371/journal.pcbi.1013534)
Supplement: S8 Fig — (DOCX) [file pcbi.1013534.s008.docx]

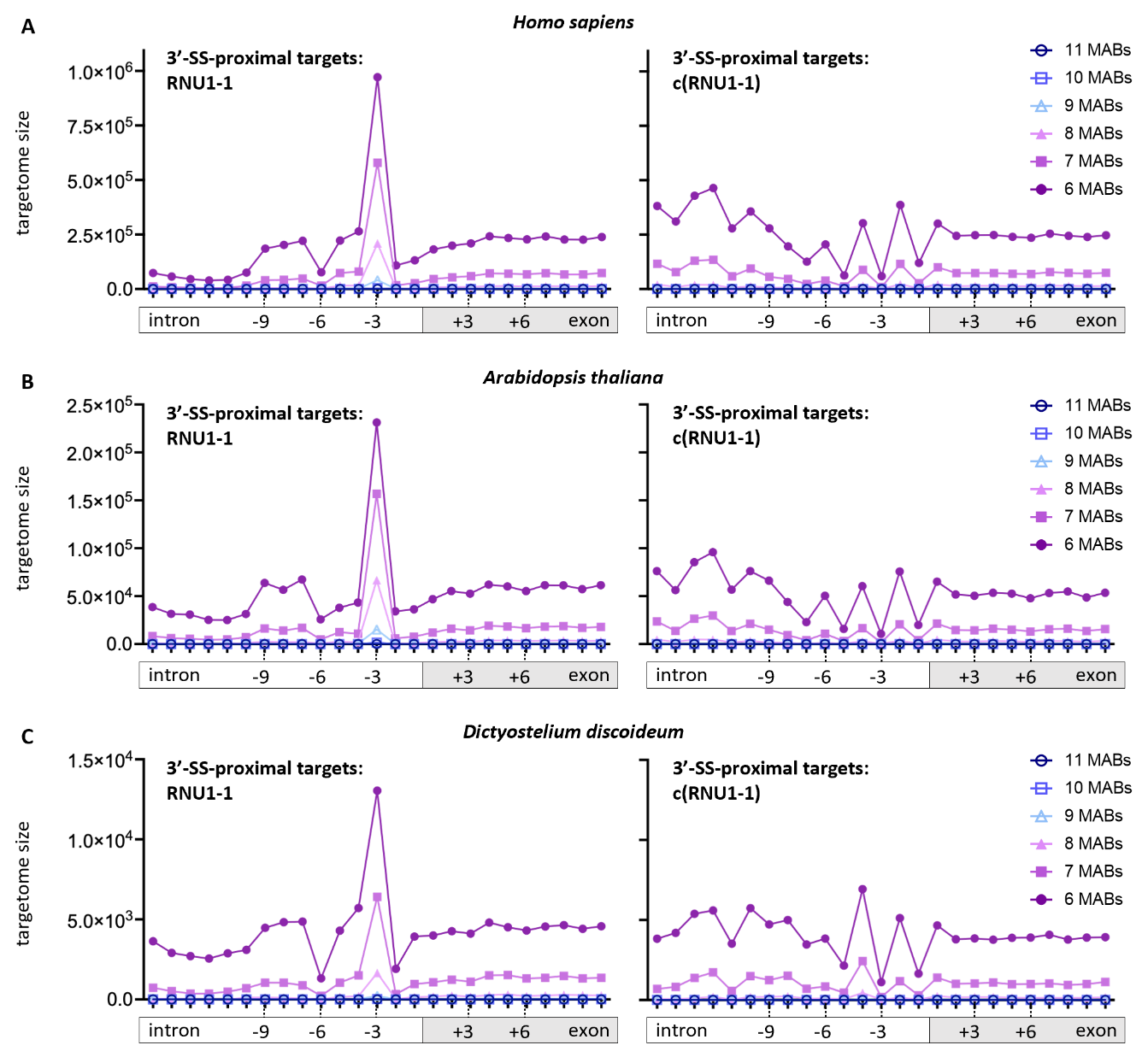


**S8 Fig.** Target distribution in proximity of 3’-SSs for the endogenous U1 (left) and c(RNU1-1) (right) in (**A**) *H. sapiens*, (**B**) *A. thaliana* and (**C**) *D. discoideum*. Target counts are shown as a function of MABs. Sites are 1 nt apart, ranging from 15 nt up- to 10 nt down-stream of the exon-intron junction (with reference to the 5’-most position of the target sequence).
