# Supplementary material for: Utargetome: A targetome prediction tool for modified U1-snRNAs to identify distal-target positions with improved selectivity
Source: PLoS Comput Biol. 2025 Sep 23;21(9):e1013534. doi: 10.1371/journal.pcbi.1013534 (PMC12527174; doi:10.1371/journal.pcbi.1013534)
Supplement: S9 Fig — (DOCX) [file pcbi.1013534.s009.docx]

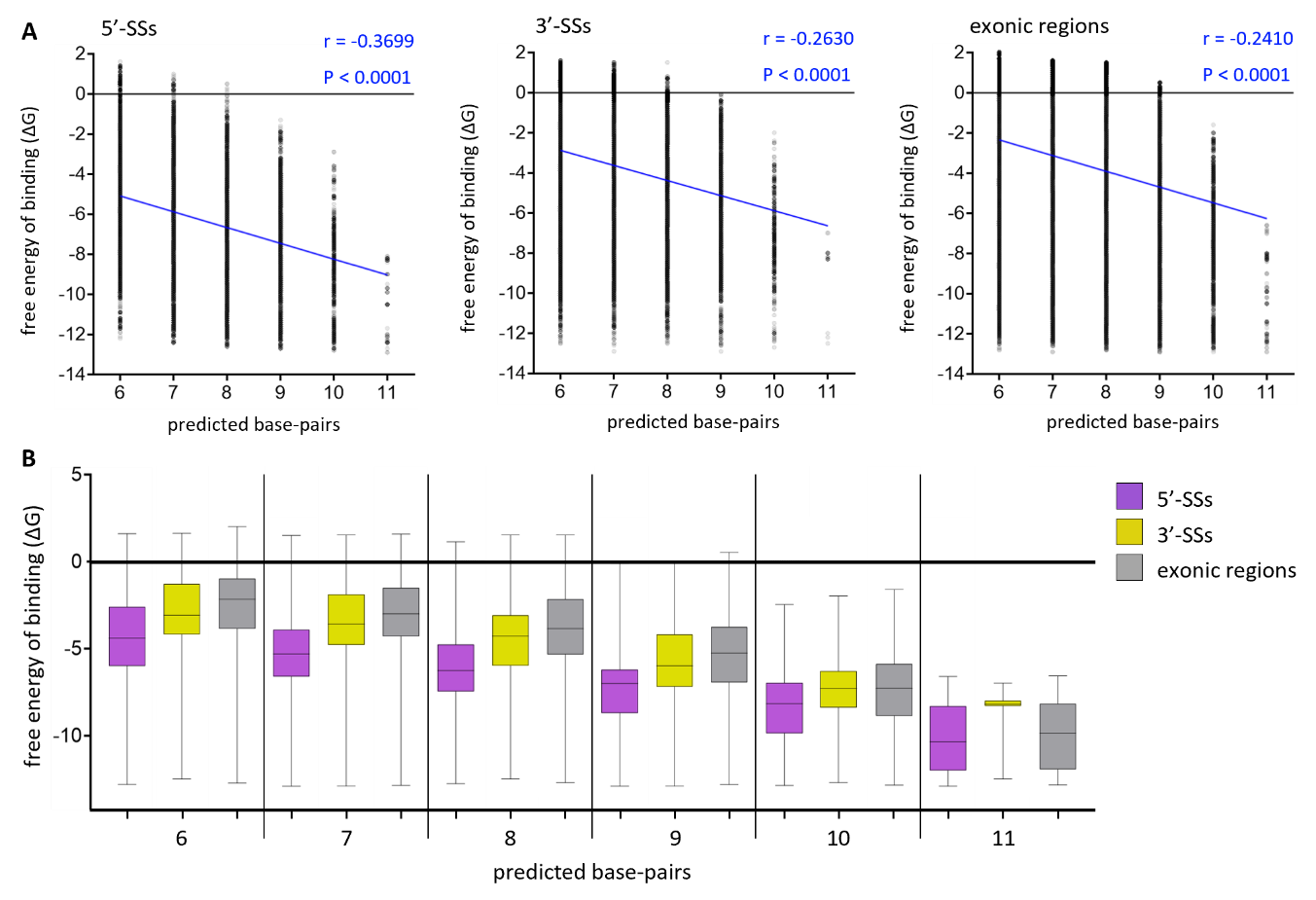


**S9 Fig.** Analysis of free energy of binding for the predicted targets of the human endogenous U1 at positions overlapping with 5’-SSs, 3’-SSs and exonic regions. (**A**) Correlation between estimated free energy of binding (ΔG) and the number of predicted base pairs is shown for each type of U1 targets (5’-SSs, 3’-SSs or exonic regions) with a fitted linear regression (blue line), along with Pearson correlation coefficient (r) and P-value. (**B**) Box blots indicating the minimum, first quartile, median, third quartile, and maximum ΔG estimated for the different types of U1 targets (5’-SSs, 3’-SSs or exonic regions), classified by the number of predicted base-pairs.
