# Supplementary material for: Utargetome: A targetome prediction tool for modified U1-snRNAs to identify distal-target positions with improved selectivity
Source: PLoS Comput Biol. 2025 Sep 23;21(9):e1013534. doi: 10.1371/journal.pcbi.1013534 (PMC12527174; doi:10.1371/journal.pcbi.1013534)
Supplement: S10 Fig — (DOCX) [file pcbi.1013534.s010.docx]

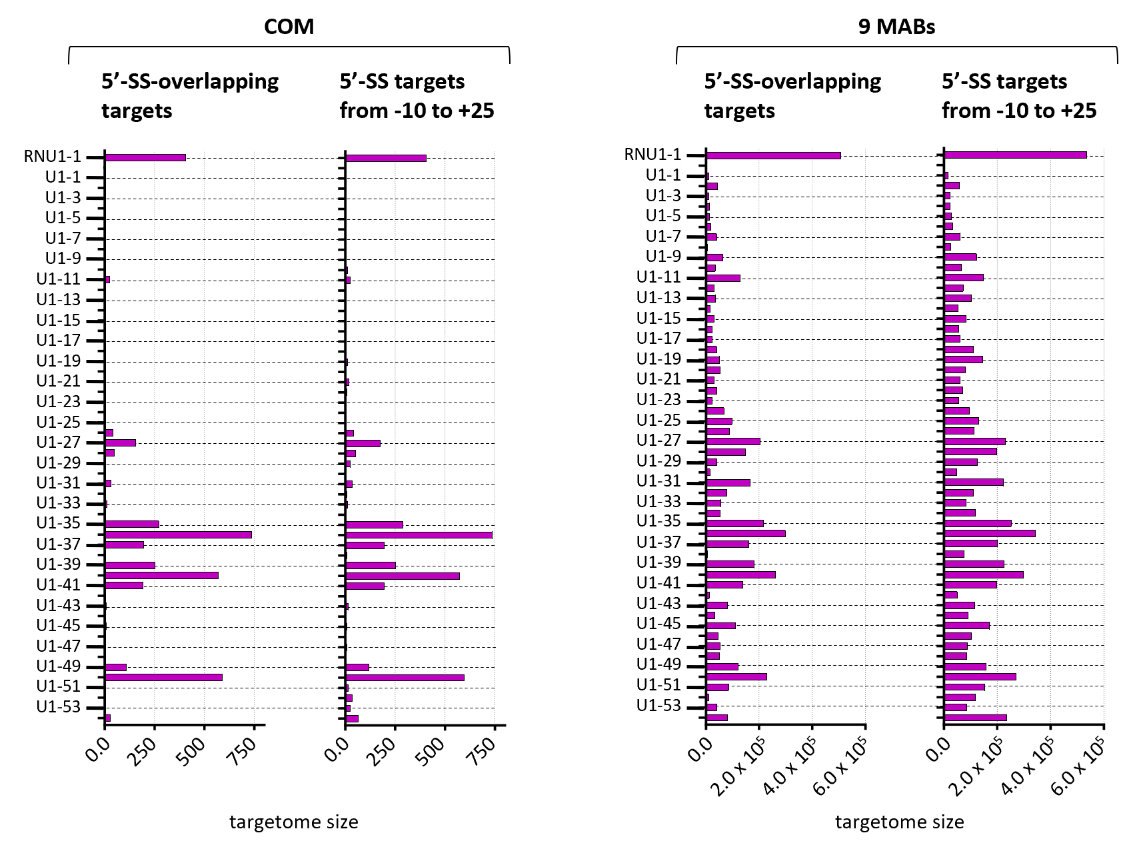


**S10 Fig.** 5’-SS targets for the 54 modified U1s. A comparison is shown between counts of targets overlapping with 5’-SSs and counts of all targets within a range from 10 nt up- to 25 nt down-stream of the exon-intron junction (with reference to the 5’-most position of the target sequence). The comparison is shown for perfectly complementary targets (COM) and for targets with 9 MABs. Refer to Fig 4A to 4C for the legend.
