# Supplementary material for: Utargetome: A targetome prediction tool for modified U1-snRNAs to identify distal-target positions with improved selectivity
Source: PLoS Comput Biol. 2025 Sep 23;21(9):e1013534. doi: 10.1371/journal.pcbi.1013534 (PMC12527174; doi:10.1371/journal.pcbi.1013534)
Supplement: S11 Fig — (DOCX) [file pcbi.1013534.s011.docx]

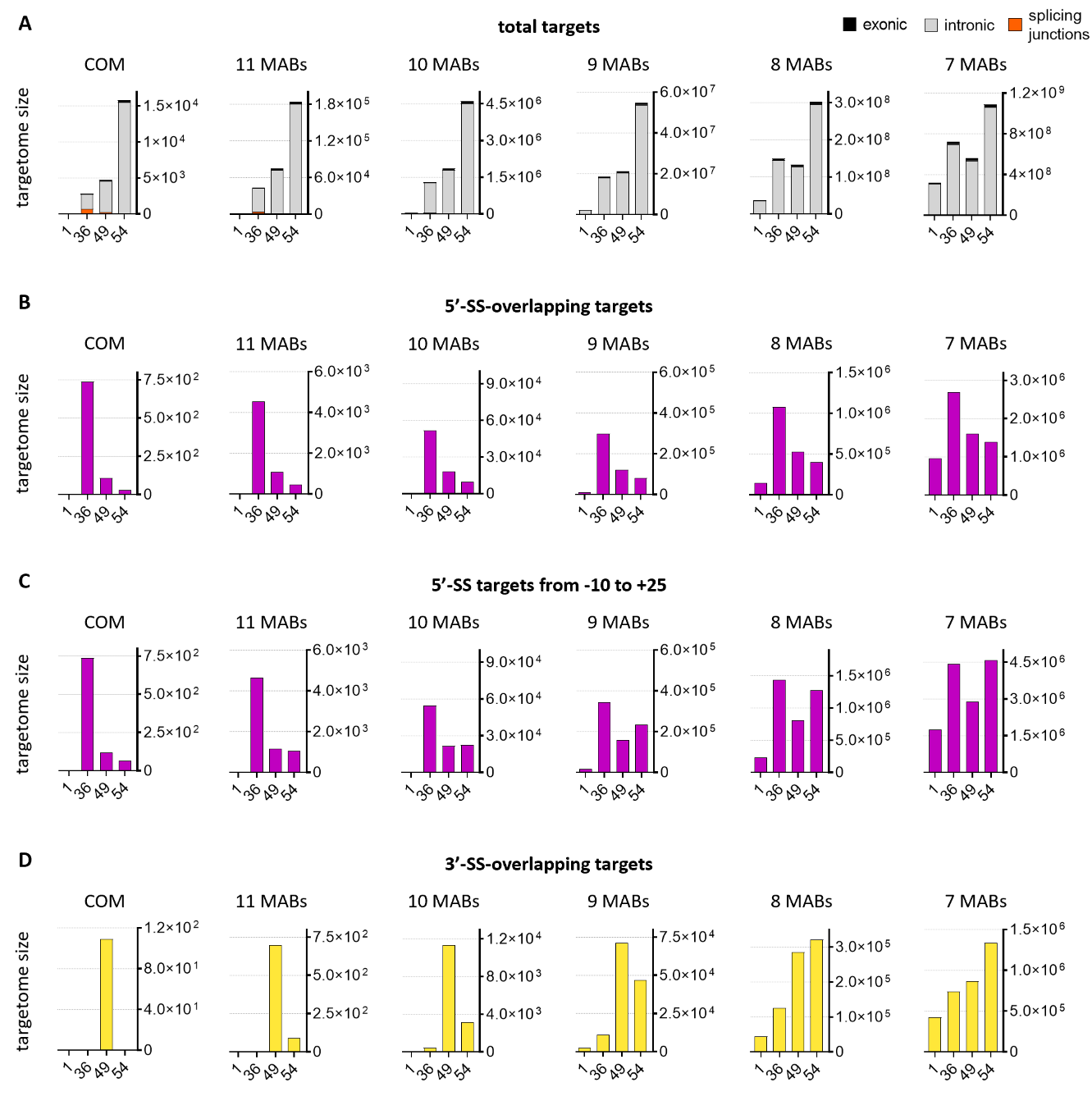


**S11 Fig.** Correlation between targetome size and decreasing complementarity (from perfect complementarity, or COM, to 7 MABs) for U1-1 (smallest targetome), U1-36 (highest 5’-SS target count), U1-49 (highest 3’-SS target count) and U1-54 (largest targetome, refer to Table S14). (**A**) Total targetome size. (**B**) 5’-SS-overlapping target count. (**C**) 5’-SS target count within a range from 10 nt up- to 25 nt down-stream of the exon-intron junction (with reference to the 5’-most position of the target sequence). (**D**) 3’-SS-overlapping target count.
