# Supplementary figures and images for: Utargetome: A targetome prediction tool for modified U1-snRNAs to identify distal-target positions with improved selectivity
Source: PLoS Comput Biol. 2025 Sep 23;21(9):e1013534. doi: 10.1371/journal.pcbi.1013534 (PMC12527174; doi:10.1371/journal.pcbi.1013534)

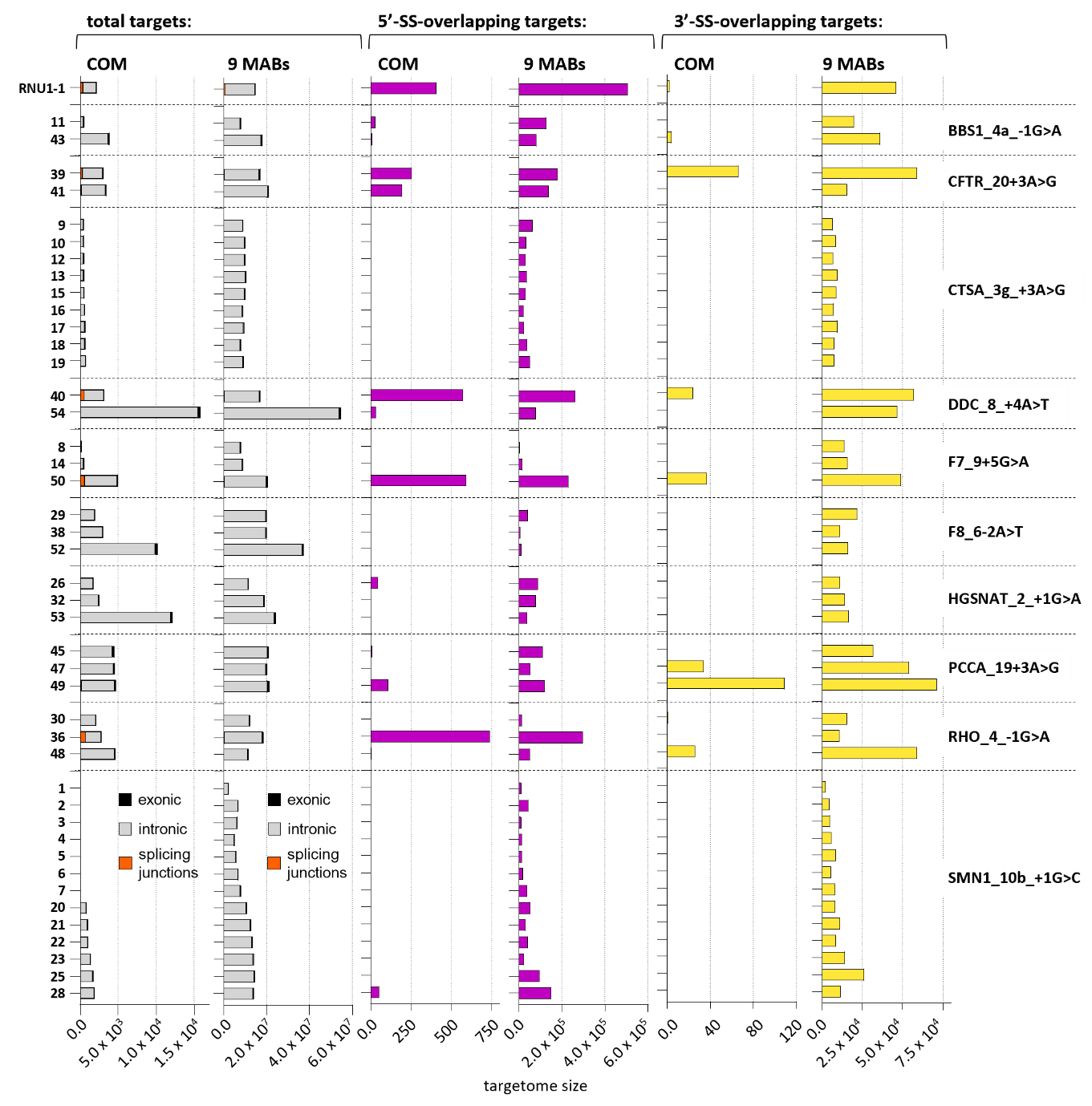


**S12 Fig.** Targetome of the 54 modified U1s grouped by target mutation. Refer to Fig 4A to 4C for the legend.

Supplement: S12 Fig — Refer to Fig 4A–4C for the legend. (DOCX) [file pcbi.1013534.s012.docx]
