# Supplementary material for: Utargetome: A targetome prediction tool for modified U1-snRNAs to identify distal-target positions with improved selectivity
Source: PLoS Comput Biol. 2025 Sep 23;21(9):e1013534. doi: 10.1371/journal.pcbi.1013534 (PMC12527174; doi:10.1371/journal.pcbi.1013534)
Supplement: S13 Fig — (DOCX) [file pcbi.1013534.s013.docx]

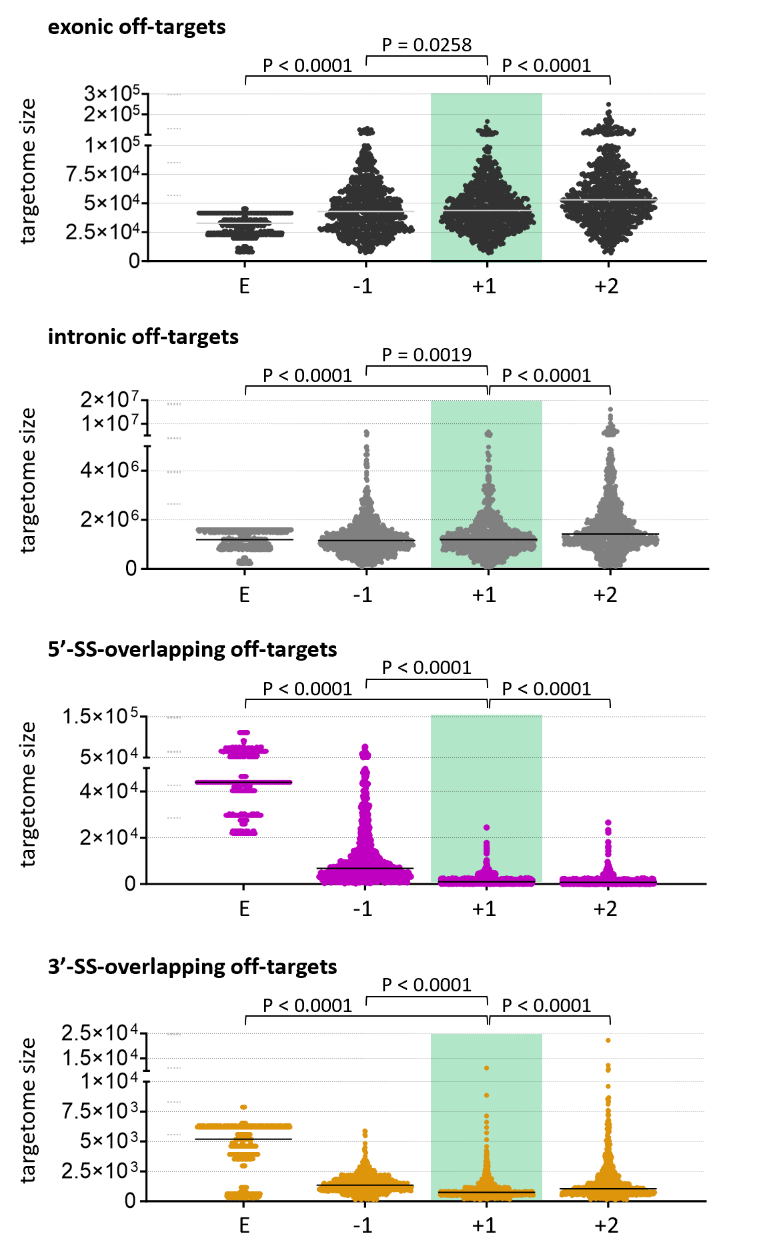


**S13 Fig.** Targetome analysis at 10 MABs for de-novo designed U1s targeting 839 unique 5’-SS mutations at selected distal positions (-1, +1 and +2). The endogenous U1 carrying a single-nucleotide adaptation to the mutation (labelled as “E”) is also included in the analysis. Counts include all targets with 10 MABs, and are shown for exonic, intronic, as well as 5’-SSs and 3’-SSs overlapping targets. The optimal target position located at +1 is highlighted in green. P-values obtained by One-Way ANOVA test are indicated for position +1 compared to the others.
