# Supplementary material for: Utargetome: A targetome prediction tool for modified U1-snRNAs to identify distal-target positions with improved selectivity
Source: PLoS Comput Biol. 2025 Sep 23;21(9):e1013534. doi: 10.1371/journal.pcbi.1013534 (PMC12527174; doi:10.1371/journal.pcbi.1013534)
Supplement: S14 Fig — (DOCX) [file pcbi.1013534.s014.docx]

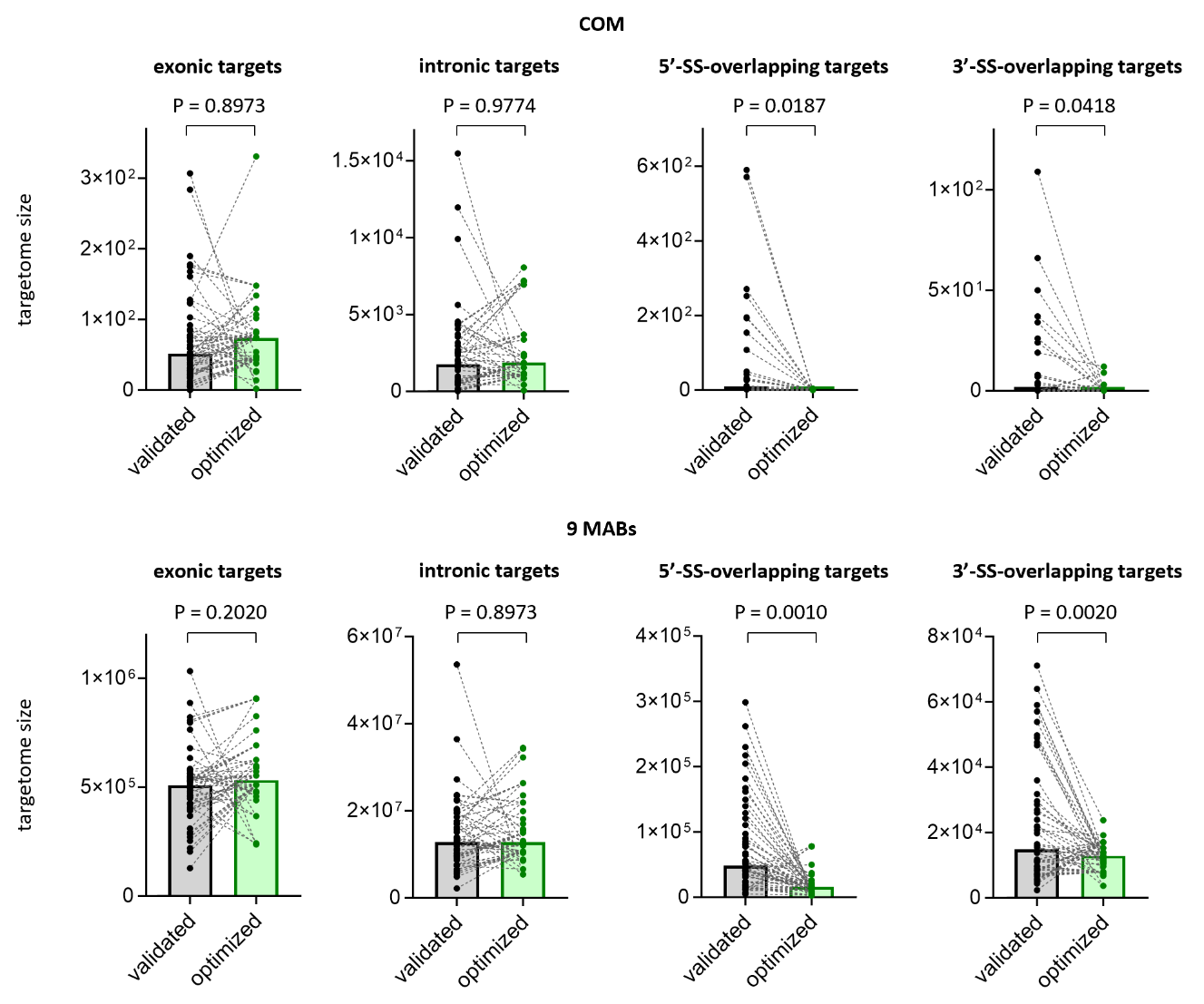


**S14 Fig.** Comparison of targetome size between modified U1s validated in literature (black) and the newly designed U1s targeting the distal position +1 as indicated in Fig 5C (green). The comparison is shown for targets with perfectly complementarity (COM, top panel) and with 9 MABs (bottom panel). Median target counts are depicted in addition to single target counts. P-values obtained by paired t-test and adjusted for False Discovery Rate are reported for each pairwise comparison.
