# Supplementary material for: Utargetome: A targetome prediction tool for modified U1-snRNAs to identify distal-target positions with improved selectivity
Source: PLoS Comput Biol. 2025 Sep 23;21(9):e1013534. doi: 10.1371/journal.pcbi.1013534 (PMC12527174; doi:10.1371/journal.pcbi.1013534)
Supplement: Attachment — Submitted filename: Response to Reviewers.docx [file pcbi.1013534.s027.docx]

*From:*

*Keng Boon Wee, PhD*

*Principal Scientist, RNA Modulations and Therapy Lab*

*Institute of Molecular and Cell Biology (IMCB), Agency for Science, Technology and Research (A*STAR), Proteos building, Republic of Singapore*

*19^th^ August 2025*

***RE: PCOMPBIOL-D-24-02008 (Utargetome: a targetome prediction tool for modified U1-snRNAs to identify distal-target positions with improved selectivity)***

Dear Bishoy and Shihua,

Thank you for giving us the opportunity to address the points raised by the reviewers and your comments. We are especially grateful to you for allowing two deadline extensions for us to complete the additional computational work and analyses to address the points thoroughly.

I have provided our point-by-point response to each comment in this letter. In addition, we have addressed all the formatting and technical items to satisfy the requirements of the journal.

Thank you for your further consideration of our revised manuscript.

Sincerely,

Keng Boon Wee, Ph.D.

**Additional Editor Comments :**

1. The function of U5 and U6 snRNAs in base-pairing with the 5’ splice site subsequent to U1 should be elaborated, ensuring that their roles are either adequately recognized or explicitly refuted about modified U1s binding to distal sites.

**Response:** We have added texts in the Discussion {R1Q1} to discuss the roles of U5 and U6 snRNAs in the context of both endogenous and modified U1s.

2. Furthermore, Figure 1 requires modification to offer a more thorough elucidation of the endogenous U1 structure, specifically highlighting the function of pseudouridines in U1 binding in addition to a clear indication of the numbering scheme.

**Response:** We have expanded the explanation of annealing configurations and position annotation in the Results {R2Q5} and Methods {R2Q5} sections to clarify how each target is generated and labeled. We have also added a new supplementary figure (now S2 Fig) that illustrates how the targetome of a given U1 is constructed. This figure shows the progressive implementation of mismatches, loops, and bulges from a fully complementary base-pairing (COM), and explains how the positional annotation of a target relative to the 5′ splice site is determined. Specifically, the target position always refers to the 5′-most nucleotide on the RNA target sequence (i.e., the 3′-most base of the U1 antisense sequence), independent of whether the annealing contains mismatches or structural features such as bulges. Therefore, a site labeled as “–3” maintains that label regardless of whether base-pairing occurs at all 11 U1 nucleotides, or if some positions are mismatched or disrupted by structural features. We hope this illustration and the additional textual clarifications now provide a comprehensive understanding of the target positioning system used in Utargetome. Additionally, we have also expanded the Discussion {R1Q2} to address the presence and functional role of pseudouridines in the 5′ end of U1-snRNA. We now highlight their known contribution to stabilizing the RNA structure and enhancing U1’s interaction with 5′ splice site, and we acknowledge the current lack of data on whether pseudouridylation occurs in modified U1s and how it may influence their activity.

3. The notion of Minimum Annealed Bases (MAB) necessitates clarification, specifically about whether it encompasses solely Watson-Crick base pairs or also considers G-U wobble pairings, which naturally arise in 5’ splice site/U1 interactions.

**Response:** We clarify that MAB considers U1:target interactions based on the minimum number of canonical Watson-Crick base pairs (A:U and G:C). In the current implementation, G:U wobble pairs are considered as mismatches because the tolerance for G:U pairing under a wide repertoire of modified U1 sequences are not known. Target sites with incidence of wobble pairs will thus have lower MABs than when wobble pairs are considered explicitly. We have provided a more detailed explanation of MABs in both the Introduction {R1Q4} and Results {R1Q4} sections, and also updated Figure 2 to illustrate how MAB-based classification is integrated into the computational pipeline.

4. The descriptions for Figures 4 and 5 need improvements to clearly highlight the major findings and facilitate a direct comparison between the expected Utargetome.

**Response:** We have added texts in the Results {R1Q5} to better explain and emphasize the biological implications of Figure 4. In addition, we have now added to Figure 4E the biological relevant exons at 9 MABs, and the annealing registers with mismatch (if any) manifested by each of the relevant exons. While we cannot ascertain the specific exons affected by U1-36 without wet experiments, the sheer number of biologically relevant exons identified from the targetome inevitably increases the probability of actual off-target events. Similarly, we have added texts to the Results {R1Q5} for Figure 5. Unfortunately, a direct comparison with RNAseq–based off-target analyses is not possible due to the limited literature [25-27]. They were either conducted in mouse tissues or did not provide the complete list of off-target events. We added a brief literature survey in the Discussion {R1Q5}.

**Reviewers' comments:**

**Reviewer #1:** This manuscript reports a new bioinformatics tool to predict the off-targets of mutant U1 snRNAs which are under evaluation for splice-correcting therapeutics, and this algorithm should improve the design of such modified U1s for high effectiveness and low off-target effects, mainly the latter. This algorithm accommodates exceptions of the target 5’ss and U1 base-pairing register, and its efficiency is supported by its ability to recognize most bona-fide human 5’ splice sites with the sequence of the endogenous U1. By analysing 54 modified U1s tested in other works, they found a range of predicted off-targets, and that targeting few nucleotides downstream of the canonical 5’ss (distal sites) shows the least predicted off-targets. This study should be useful to guide the assessment of the off-targets of modified U1s, to improve their design for therapeutic applications. My comments below are minor yet necessary for improving this manuscript:

1. The role of the U5 and U6 snRNAs in the base-pairing to different portions of the 5’ splice site after U1 should be better described as this is now too succinct. While the binding of modified U1s to the identified distal sites should not be followed by U5 and U6 at these sites, authors can ignore their contribution for the mode of action of modified U1s, yet this point needs to be addressed properly.

**Response:** We appreciate the suggestion to discuss the roles of U5 and U6 snRNAs in the context of both endogenous and modified U1s. We added the texts in the Discussion {R1Q1}.

2. Figure 1 shows the endogenous U1 with its 5’ end. Since the two pseudouridines in the wild-type U1 contribute to the binding of 5’ splice sites, these modified nucleotides should be mentioned more extensively in the text, with 1-2 more citations.

**Response:** We appreciate this important point and have expanded the Discussion section {R1Q2} on the presence and functional role of pseudouridines with additional references.

3. Considering that the base-pairing between the 5’ end of U1 and the 5’ splice site is an RNA:RNA interaction, there are algorithms to estimate the free energy (deltaG) of binding, which often correlates with splicing efficiency. Likewise, the deltaG of modified U1s can also be estimated for off targets. Overall, authors would need to compare their Utargetome algorithm with the free-energy of binding.

**Response:** In the field of antisense oligonucleotides particularly splice-switching oligonucleotides (SSOs), which bind to nascent mRNA via Watson Crick bonding rules and effect steric blockage to RNA-binding proteins involved in splicing, we agree that ΔG is an important parameter for SSO efficiency in splice modulation. There is however a range of ΔG that is optimal for SSO binding beyond which splicing efficiency will be diminished. As the SSO sequence must be 100% complementary to its target mRNA sequence, one resorts to specific chemical modifications on the SSO to attain the optimal ΔG.

On the other hand, U1snRNAs work for non-complementary sequences and most contrastingly, their mechanistic mode of action is enzymatic in nature since they initiate a cascading events leading to spliceosome formation. As a result, splicing efficiency is not easily inferable from ΔG of binding between U1 and mRNA, unlike SSO whose mode of action is non-enzymatic and thus splicing efficiency is more correlated with its binding kinetics. Because of the infidelity of U1 binding, our study focuses on predicting the targetome but does not attempt to rank or discriminate these target sites by their MABs (minimum annealing bases between the U1 and mRNA) nor by their ΔG.

Nonetheless, we have extensively evaluated ΔG for the targetome of the human endogenous U1 at canonical 5′ splice sites, 3’ splice sites and exonic positions from MABs 11 to 6. Two main observations can be noted. The first is, as expected, ΔG is inversely correlated with MABs (Figure S11A), which is not an appropriate indicator of U1 binding likelihood given U1’s infidelity. Secondly, ΔG at 5’SS are generally lower than at the other two positions (Figure S11B), which may suggest U1 preferential binding to 5’SS sequences, a well-established fact. We have added the results to the Results section {R1Q3}, a new Figure S11, Methods section {R1Q3} and discussed in the Discussion {R1Q3}.

In conclusion, our approach in using annealing registers has the advantage to provide information on the exact annealing structure formed by every target in the targetome. Furthermore, at low MABs, it is computationally not straightforward for a ΔG-based approach to identify all possible target sites without the explicit consideration of mismatches, loops and bulges.

4. We are also missing a more detailed definition of MAB as minimum annealed bases. Do MABs only correspond to Watson-Crick base pairs? Do MABs allow wobble base pairs (G-U) which also occur in 5’ splice site / U1 helices?

**Response:** We apologize for the ambiguity caused by the current definition of MAB. We clarify that MAB considers U1:target interactions based on the minimum number of canonical Watson-Crick base pairs (A:U and G:C). In the current implementation, G:U wobble pairs are considered as mismatches because the tolerance for G:U pairing under a wide repertoire of modified U1 sequences are not known. Target sites with incidence of wobble pairs will thus have lower MABs than when wobble pairs are considered explicitly. We have provided a more detailed explanation of MABs in both the Introduction {R1Q4} and Results {R1Q4} sections, and also updated Figure 2 to illustrate how MAB-based classification is integrated into the computational pipeline.

5. The data shown in Figure 4 and its in-text description in Results needs better explanations, and the main conclusions emphasized, to make sure the main point of the work is clear. How does the predicted Utargetome compare to experimentally derived off-targets reported in other works? Same notion applies to Figure 5.

**Response:** We thank the reviewer for highlighting the useful suggestions to improve the manuscript. We have added texts in the Results section {R1Q5} to better explain and emphasize the biological implications of Figure 4. In addition, we have now added to Figure 4E the biological relevant exons at 9 MABs, and the annealing registers with mismatch (if any) manifested by each of the relevant exons. While we cannot ascertain the specific exons affected by U1-36 without wet experiments, the sheer number of biologically relevant exons identified from the targetome inevitably increases the probability of actual off-target events. Similarly, we have added texts to the Results section {R1Q5} for Figure 5. Unfortunately, a direct comparison with RNAseq–based off-target analyses is not possible due to the limited literature [25-27]. They were either conducted in mouse tissues or did not provide the complete list of off-target events. We added a brief literature survey in the Discussion section {R1Q5}.

**Reviewer #2**: The manuscript by Pigini and colleagues describes a bioinformatic investigation into the specificity of splicing changes induced by the expression of U1 snRNAs that are designed to rescue a splicing event that has been compromised by mutations in the sequence recognised by wild-type U1 snRNA. Using previous evidence for the variety of ways in which U1 snRNA can base-pair with 5'SS, they first look at possible targets in the transcriptome for wild-type U1 snRNA and then analyse the possible targets for 54 modified U1 snRNAs that have been shown elsewhere to restore splicing to defective sites. The goal of the work is to establish whether there is an optimum position near the target 5' splice site to which a rescuing U1 snRNA should be directed so as to minimize off-target effects.

1. The first part of the Results is devoted to establishing that the method is valid by showing that it correctly identifies sites at which the wild-type U1 snRNA might bind or act. The evidence given is that known 5'SS are identified (Fig. 3C, etc.), with U1 snRNA predicted to bind predominantly at the correct position (Fig. 3D). Most of the predicted sites of interaction (99.9%, from Figures 3A and B) were not known 5'SS, but no attempt was made to investigate whether they were in fact sites of base-pairing interactions. This could have been established by the use of existing data from psoralen cross-linking.

**Response:** We acknowledge the important point raised by the reviewer on whether predicted non-5’SS target sites are indeed sites of base-pairing interactions with U1. We also agree that transcriptome-wide RNA-RNA interaction datasets obtained through psoralen-based crosslinking and ligation methods (e.g., PARIS, SPLASH, LIGR-seq) can in principle be used to address it. Unfortunately, we are not able to find existing data. In fact, there is only a handful of psoralen-based papers reporting U1 interactions with a specific 5’SS on minigene systems that were expressed exogenously; PMIDs 12486009, 1411506 and 8384583, and <https://doi.org/10.1038/npre.2009.4119.1>. Nonetheless, we have added text in the Discussion {R2Q1} highlighting the important point raised by the reviewer. We hope our work will spur new interest in the psoralen field to study transcriptome-wide U1 targetome.

2. The second part of the results is based on an analysis of 54 modified U1 snRNAs that have been used previously for rescuing 5'SS mutations. After some analysis of the distribution of potential hits according to the particular sequence tested and the number of complementary base-pairs, the authors analysed RNAseq datasets to identify exons that might be affected by U1-36, which was designed to rescue a specific retinopathy. They identified 39 exons with fully complementary 5'SS that might be affected by U1-36. However, no data were shown to show that these exons had been affected by U1-36. More to the point, no use was made of the results obtained by analysing all the possible alignments and base-pairing configurations in the manner described earlier.

**Response:** Unfortunately, a direct comparison with RNAseq–based off-target analyses is not possible due to the limited literature [25-27]. They were either conducted in mouse tissues or did not provide the complete list of off-target events. We added a brief literature survey in the Discussion {R2Q2}.

We workaround this issue by thoroughly interrogating 5’-SS target sites in exons expressed in human retinal, given that they are the most probable binding sites for U1. We further filter exons with low inclusion levels (PSI < 0.5 , i.e. they are spliced-out in more than 50% of the coding transcripts, estimated from 8 independent RNA-Seq datasets), since a significant increase in PSI induced by U1-36 is likely to have biological implications. We added texts in the Results section {R2Q2} to better explain and emphasize the biological implications of Figure 4. In addition, we have now added to Figure 4E the biological relevant exons at 9 MABs, and the annealing registers with mismatch (if any) manifested by each of the relevant exons. While we cannot ascertain the specific exons affected by U1-36 without wet experiments, the sheer number of biologically relevant exons identified from the targetome inevitably increases the probability of actual off-target events.

3. The final part of the results addresses the question about the optimal position, from the point of view of minimizing binding to non-target sites. Questions about the optimal site from the point of view of the mechanisms involved were not considered. Three modified U1 genes, shown to rescue splicing, were considered for each of two genes and shown to exhibit a wide range of potential off-target binding sites. The authors then analysed the potential 'targetome' for over thirty thousand U1 snRNAs that would be candidates for rescuing 839 5' splice site mutations, although the analysis was restricted to only perfectly complementary targets. The results suggested that U1 annealing from position +1 (to + 11) of the 5' splice site might be optimal. This result was taken further by demonstrating that the 54 modified U1 snRNAs studied earlier might have been more selective if the +1 design had been used.

**Response:** The mechanism of U1 distal targeting is well-established and we have cited the relevant publication “*Singh and Singh. A novel role of U1 snRNP: Splice site selection from a distance. Biochim Biophys Acta Gene Regul Mech. 2019;1862(6):634-42*”. Please kindly point to us any reference that we have missed and we will promptly cite them in the manuscript. Grounded by this mechanism, our study is motivated by the phenomenon that sequences at distal positions are generally less conserved than at canonical 5’-SSs, and several distal-targeting U1s amongst the 54 modified U1s (Figure 5 and S20 Table) are reported to be efficacious.

We have attempted to determine the targetome at non-complementary MABs for each of the 30,204 modified U1s designed for rescuing 839 5' splice site mutations. Due to the huge computational resources required, we have only managed to obtain the targetome for modified U1s at targeting positions -1, +1 and +2 at 10 MABs. Although the targeting positions analysed are very limited, U1 at +1 targeting position still has the least 5’-SS and 3’-SS targets; we have added texts in the Results section {R2Q3} and Figure S23. If we infer from the observation that the pattern of target counts (relative selectivity) among the 54 published U1s is generally similar between perfect complementarity and 9 MABs (Figure 4A, 4B and 4C), the superior selectivity of 30,204 U1s at +1 targeting position at perfect complementarity is likely retained at 9 MABs.

4. The practicality of the approach illustrated in Figure 2 for assessing rescue U1s is not evident from the results, since two critical analyses looked only at perfectly complementary matches (Figures 4E and 5C). The other limitation is the failure to have examined experimental data to test whether there is a correlation between the predicted targetome and actual 5' splice sites affected by the rescue U1s or sites of contacts made in the transcriptome by the rescue U1s. These points should be discussed.

**Response:** We thank the reviewer for the useful suggestions to improve the Discussion section. We have expanded the Discussion {R2Q4} to discuss the use of psoralen-based crosslinking techniques and RNAseq–based off-target data, both of which are currently not available, to validate respectively the targetome and actual 5'-SS affected by the U1s, and sites of interactions made in the transcriptome by the U1s.

We think the approach illustrated in Figure 2 is practical to implement for rescuing one mutation for which a set of 35 modified U1s are designed by walking along their target transcripts, from 10 nt up- to 25 nt downstream of the exon-intron junction (Figure 5B). We agree that it is however not practical to implement simultaneously for 30,204 modified U1s designed for rescuing 839 5' splice site mutations. We envisage that users are likely to work on one or several mutations at a time, which is practical to implement as we have demonstrated by expanding the analyses to 9 MABs for the 54 published U1s (Figure 4).

Minor points:

5. The manuscript would be improved by a diagram in Figure 1 that show very clearly what the numbering system means. I infer that, where there is fully complementary base-pairing, -3 or +1 means the position in the canonical splice site to which nucleotide 11 of U1 snRNA base-pairs. However, if, with a site that would normally by designated as -3 (the wild-type U1 alignment), U1 nucleotides 9, 10 and 11 did not base-pair, would the site in that alignment still be referred to as -3, or would it now be +1? Some illustrations of the various possibilities are needed, especially when considering bulges or loops.

**Response:** We thank the reviewer for the useful suggestion. We have expanded the explanation of annealing configurations and position annotation in the Results {R2Q5} and Methods {R2Q5} sections to clarify how each target is generated and labeled. To address the Reviewer’s request directly, we have added a new supplementary figure (now S2 Fig) that illustrates how the targetome of a given U1 is constructed. This figure shows the progressive implementation of mismatches, loops, and bulges from a fully complementary base-pairing (COM), and explains how the positional annotation of a target relative to the 5′ splice site is determined. Specifically, the target position always refers to the 5′-most nucleotide on the RNA target sequence (i.e., the 3′-most base of the U1 antisense sequence), independent of whether the annealing contains mismatches or structural features such as bulges. Therefore, a site labeled as “–3” maintains that label regardless of whether base-pairing occurs at all 11 U1 nucleotides, or if some positions are mismatched or disrupted by structural features. We hope this illustration and the additional textual clarifications now provide a comprehensive understanding of the target positioning system used in Utargetome.
